# Supplementary material for: CPSign: conformal prediction for cheminformatics modeling
Source: J Cheminform. 2024 Jun 28;16:75. doi: 10.1186/s13321-024-00870-9 (PMC11214261; doi:10.1186/s13321-024-00870-9)
Supplement: Supplementary file 1 — Additional file 1: PDF file with Supplemental information, including three large tables and evaluation figures for the individual datasets. [file 13321_2024_870_MOESM1_ESM.pdf]

Supplemental information

CPSign - Conformal Prediction for Cheminformatics Modeling

Staffan Arvidsson McShane, Ulf Norinder, Jonathan Alvarsson, Ernst Ahlberg, Lars Carlsson, and Ola Spjuth

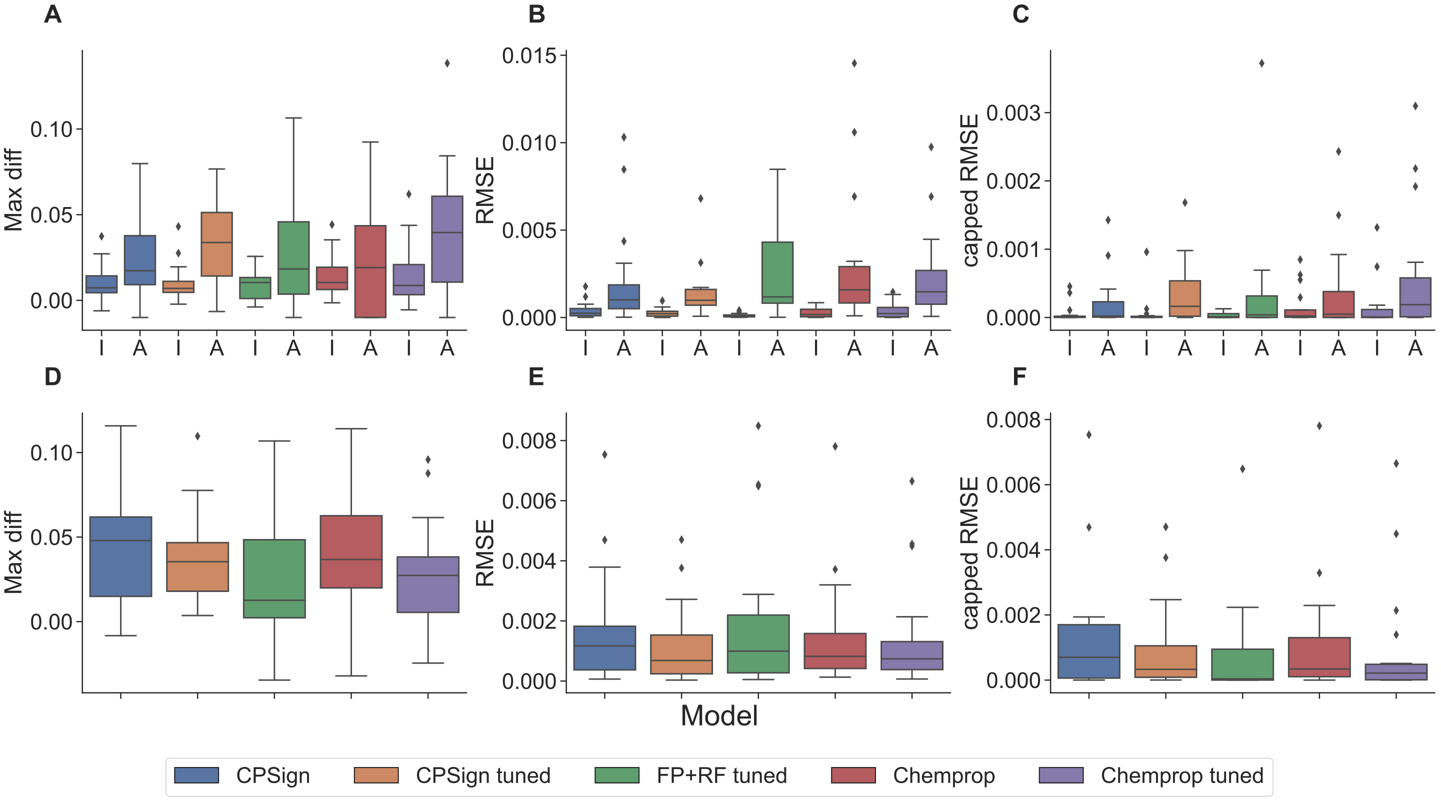

Figure 1: Calibration results aggregated for all evaluated methods and datasets, panels A-C for classification models, and panels D-F for regression models. For classification the calibration is analyzed independently per class (I: inactive, A: active), where the active class is the minority class for all datasets (Table 1, main article). The classification values were based on 30 significance levels (0.01,0.02,...,0.3) whereas the regression ones were based on six levels (0.05,0.1,...,0.3). Panel A and B display "max diff" corresponding to the expression $\max_{\varepsilon}\{error\_rate_{\varepsilon} - \varepsilon\}$, i.e. the signed difference of error-rate and significance level, where a negative value corresponds to the error rate being smaller than the significance level across all tested significance levels, and a positive value means that the error rate exceeded the significance level with at most that difference (smaller values are preferable). Panel B and E display the root mean squared error (RMSE) between the significance level and the error rate (smaller values are preferable). Panels C and F display the "capped" RMSE, in which the error rate is capped at the significance level if it is lower than the significance level (for every evaluated significance level), so that over-conservative predictions (i.e. lower error rate than required) do not contribute to a higher RMSE.

Table 1: Summary of some the features and available configurations within CPSign. Bold faced words are the default for the given row/item in the table. Further note that all of these, save from the predictor type, can be extended and injected with custom implementations. Further note that the item "Data transformations" list types of transformations, and there can be several implementations to chose from from each type.

**Item**	**Options**				
Predictor type	**ICP/ACP**	TCP	Venn-ABERS		
Classification scorer models	**LinearSVC**	C_SVC	NuSVC	LogisticRegression	
Regression scorer models	**LinearSVR**	EpsilonSVR	NuSVR		
Classification nonconf metrics	**ND2H**[a]	PD2H[b]	InverseProbability	Probability Margin	
Regression Nonconf metrics	**LogNormalized**	Normalized	AbsDiff	SignedNormalized	
P-value calculation	Standard	**Smoothed**	Linear interpolation	Spline interpolation	
Data splitting	**Random**	RandomStratified	Folded	FoldedStratified	PreDefined
Data transformations	Duplicate resolver	Filters	Imputation	Feature selection	Feature scaling
Descriptors	**Signatures**	ECFP	UserSupplied	CDK descriptors	

[a] ND2H: Negative Distance to hyperplane
[b] PD2H: Positive Distance to hyperplane

Table 2: Performance ranking of the modeling methods in the comparison. The "Top" column shows the number of datasets that each method produced the most efficient predictions, whereas the "Rank" column displays the sum of ranks across all datasets. The best value in each column is displayed in bold. OF: Observed Fuzziness.

	**Classification**								**Regression**					
	**OF**		**Average C**						**Prediction interval width**					
			$\varepsilon = 0.1$		$\varepsilon = 0.2$		$\varepsilon = 0.3$		$\varepsilon = 0.1$		$\varepsilon = 0.2$		$\varepsilon = 0.3$	
	**Top**	**Rank**	**Top**	**Rank**	**Top**	**Rank**	**Top**	**Rank**	**Top**	**Rank**	**Top**	**Rank**	**Top**	**Rank**
CPSign	**5**	44	**7**	**39**	**5**	**40**	4	48	0	56	1	53	3	53
CPSign tuned	**5**	**39**	2	46	3	43	4	**40**	**12**	**29**	**11**	**32**	**7**	**31**
FP+RF tuned	1	62	2	65	2	66	0	64	2	72	1	73	2	75
Chemprop	2	44	2	40	3	44	**5**	**40**	1	62	3	58	3	56
Chemprop tuned	3	51	3	50	3	47	3	48	3	51	2	54	3	55

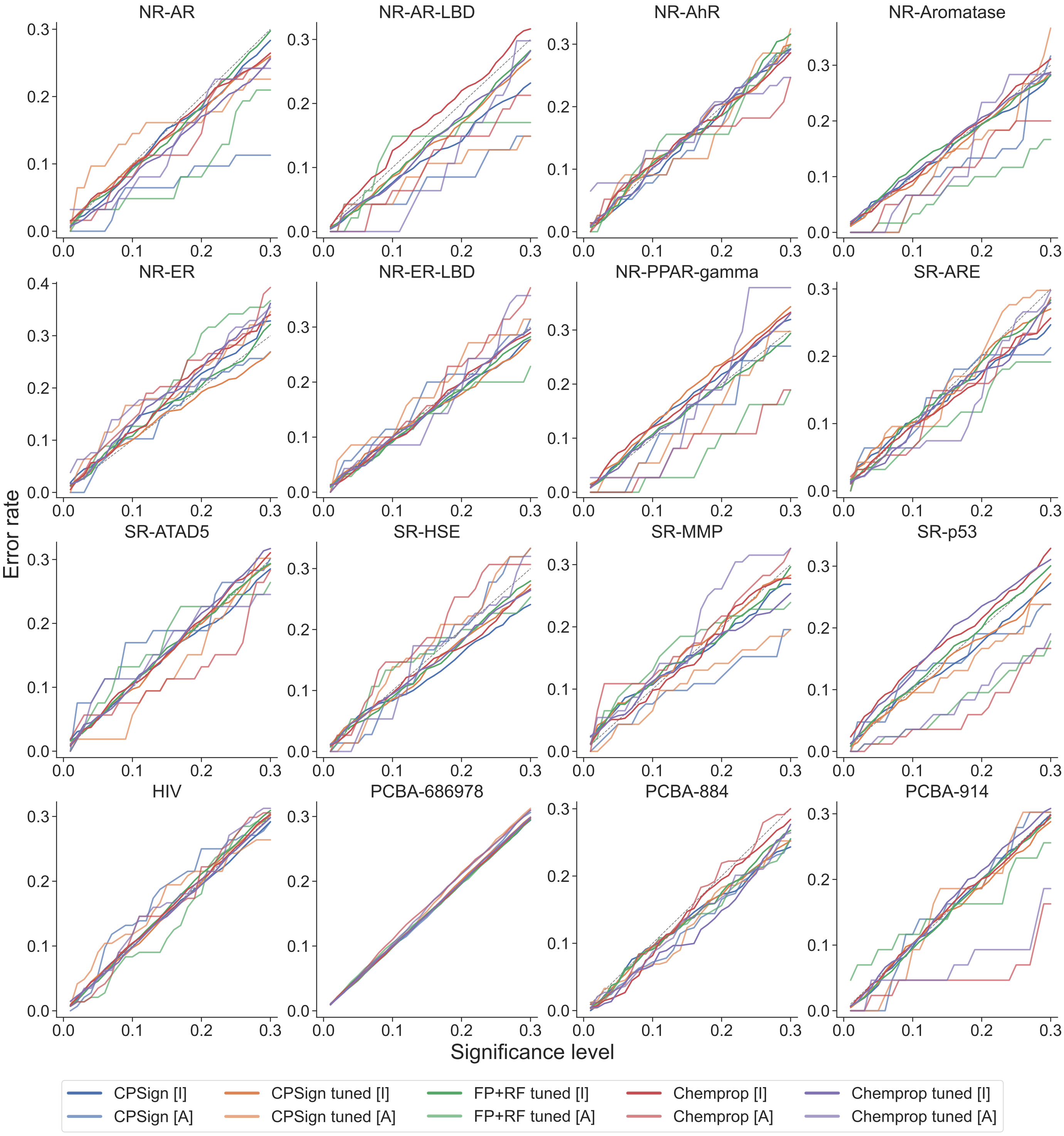

Figure 2: Calibration curves for the classification datasets, showing one curve for each class (I: inactive, A: active). The active class is the minority class for all datasets, displaying worse calibration, which can be more easily seen in the aggregation in Figure 1. All calibration curves were evaluated from 0.01,0.02,...,0.3.

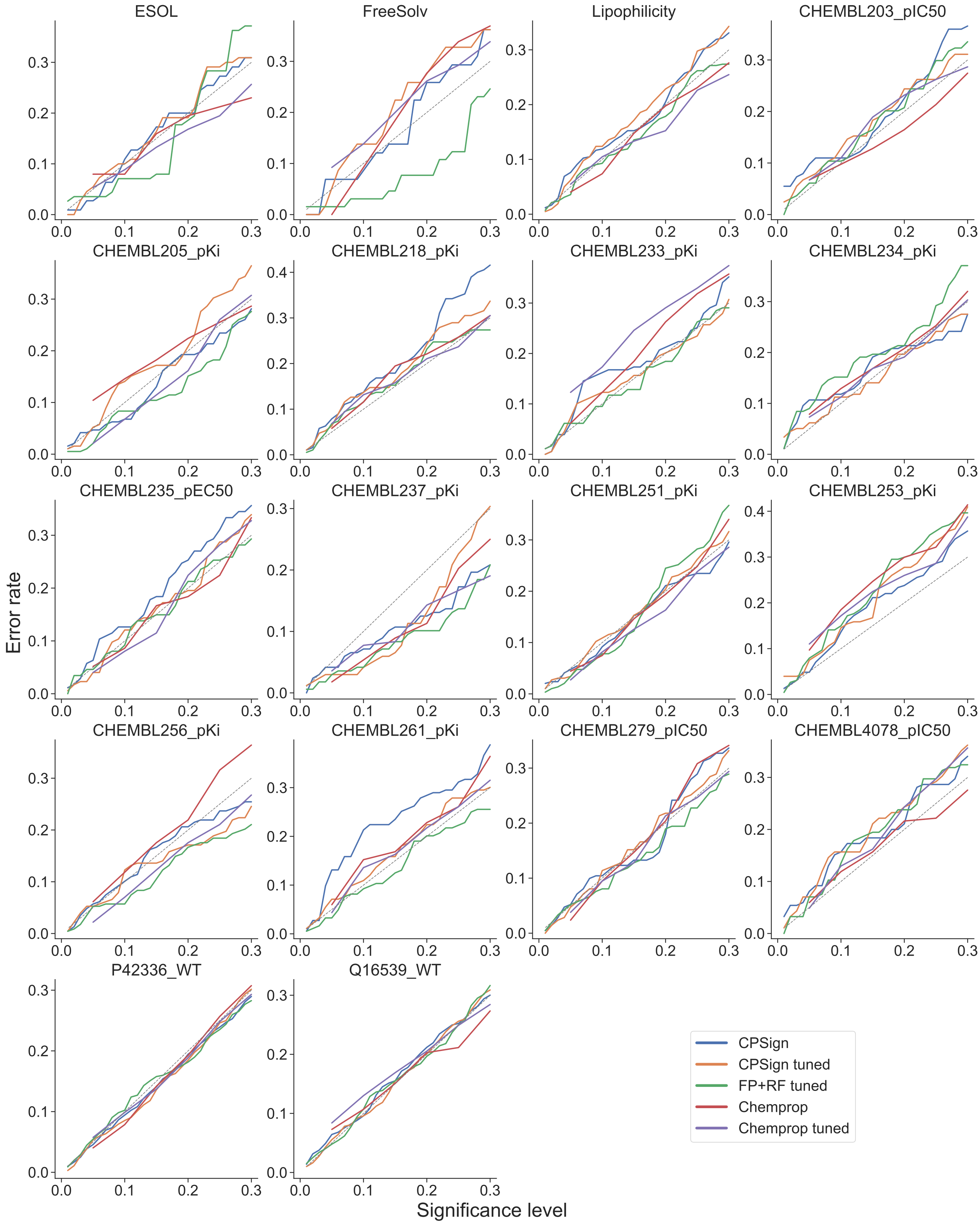

Figure 3: Calibration curves for the regression datasets. Note that the Chemprop methods were only evaluated using the six significance levels 0.05,0.1,...,0.3, whereas the other methods used significance levels 0.01,0.02,...,0.3.

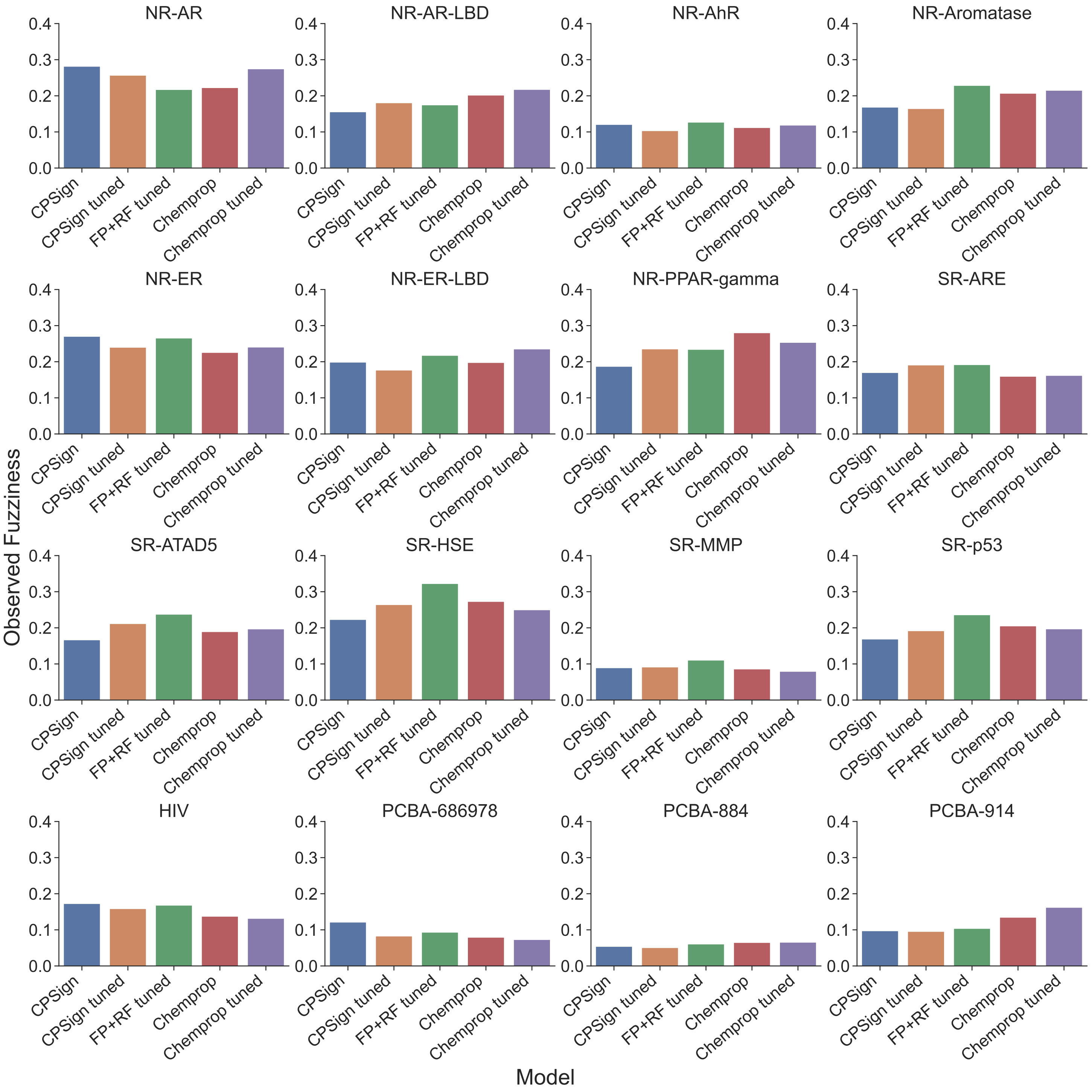

Figure 4: Comparison of the Observed Fuzziness (OF) of the evaluated methods for all sixteen datasets. A lower OF score is preferable. Larger differences can be seen when analyzing the datasets individually, and each modeling method produces the best predictions for at least one datase (see Table 2).

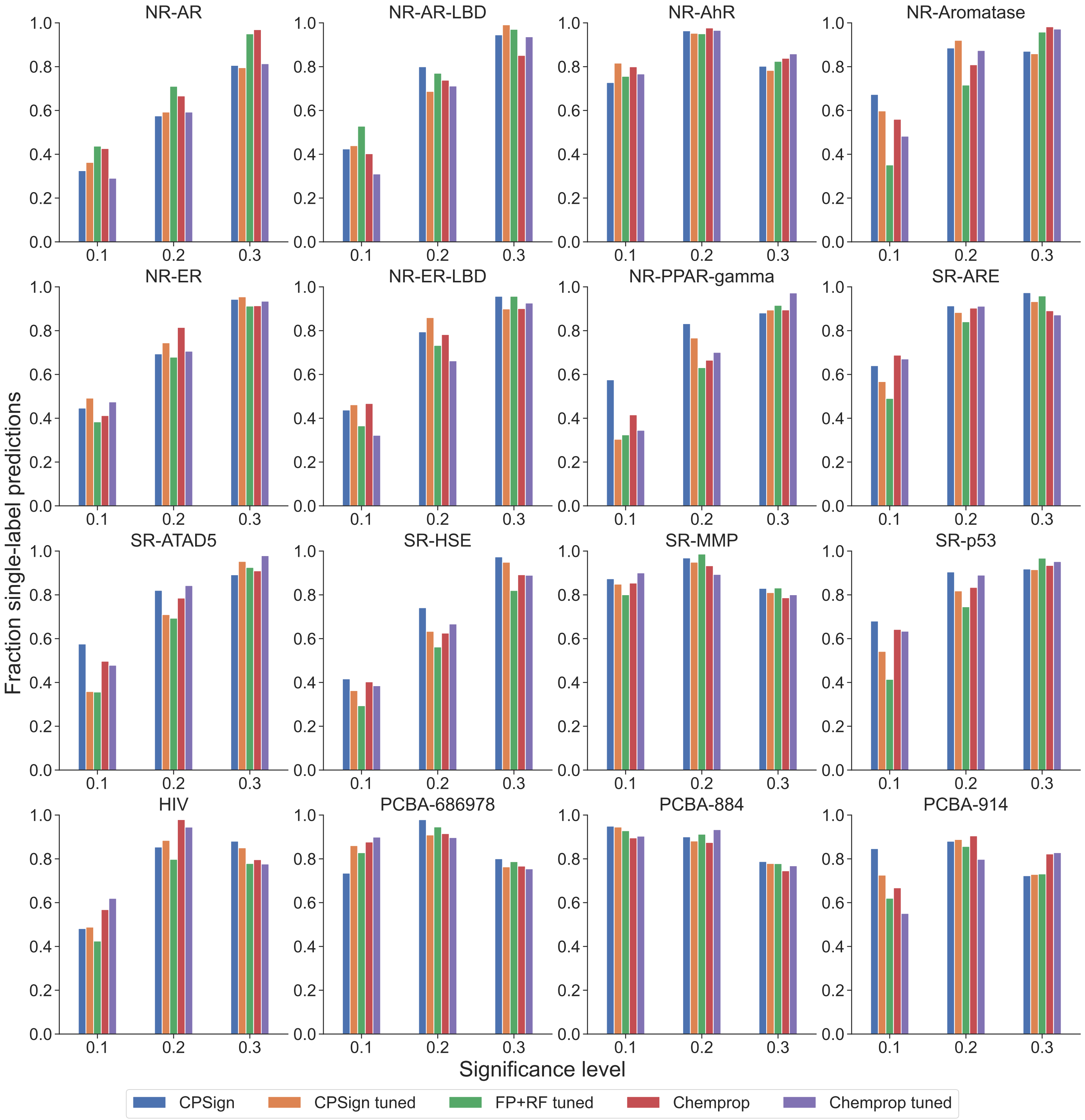

Figure 5: Median fraction of single-label predictions for the evaluated methods for all sixteen datasets, based on the three significance levels 0.1, 0.2 and 0.3 (corresponding to confidence levels 90 %, 80 % and 70 %, respectively). A higher fraction is preferable. Similarly as Figure 4 the prediction results display larger differences than the aggregated results, with different methods being the top-performing one.

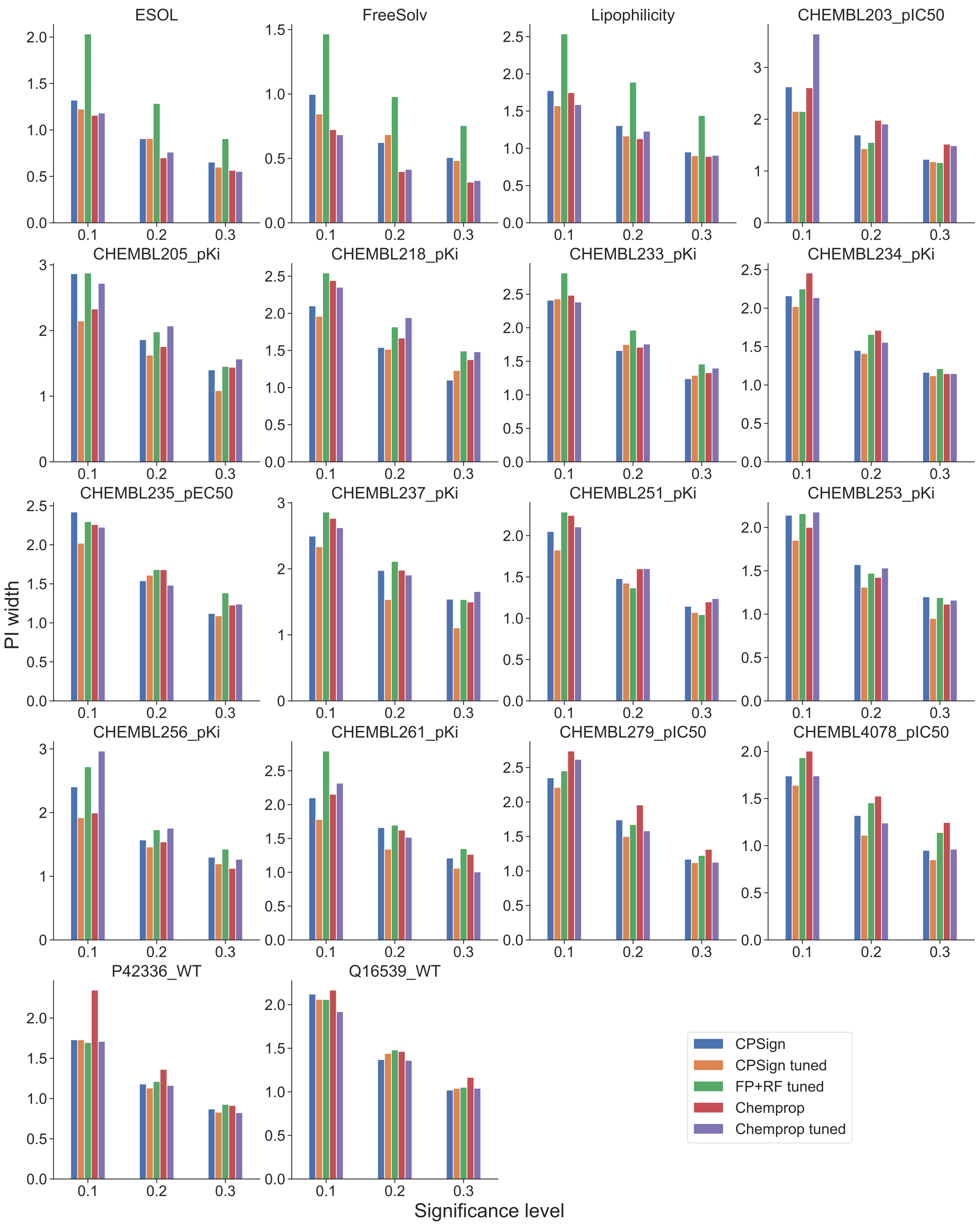

Figure 6: Median prediction interval (PI) width for all regression datasets, for significance levels 0.1, 0.2 and 0.3 (corresponding to confidence levels 90 %, 80 % and 70 %, respectively). A lower value is preferable (i.e. tighter prediction intervals).

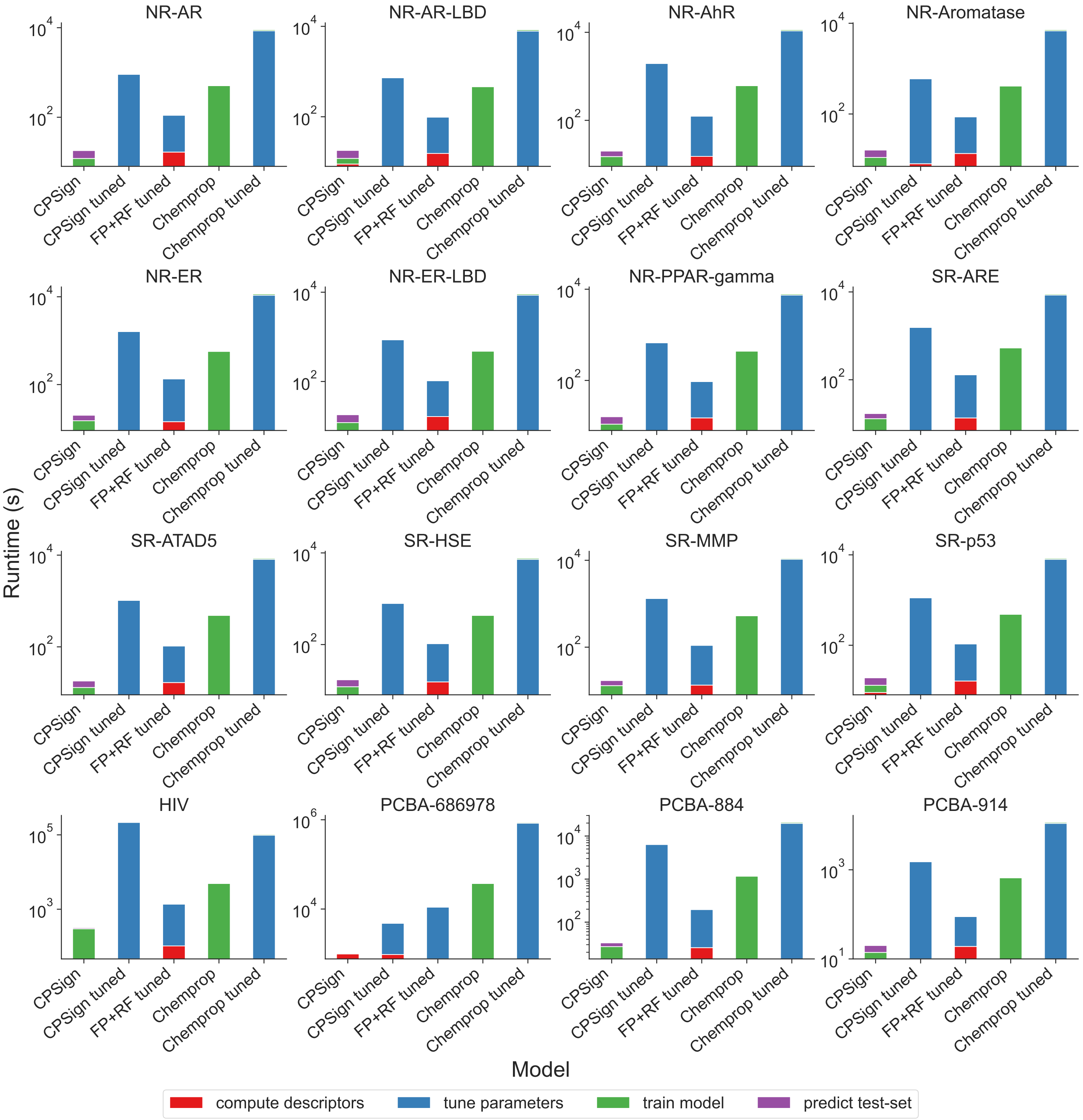

Figure 7: Runtime comparison for individual classification datasets, using a logarithmic y-axis. The relative runtimes are consistent across all runs, except the PCBA-686978 dataset (where CPSign and CPSign tuned were run with linear SVM kernels). Note that the two Chemprop methods do not contain a separate step for computing descriptors, which instead is included in the tuning and training steps.

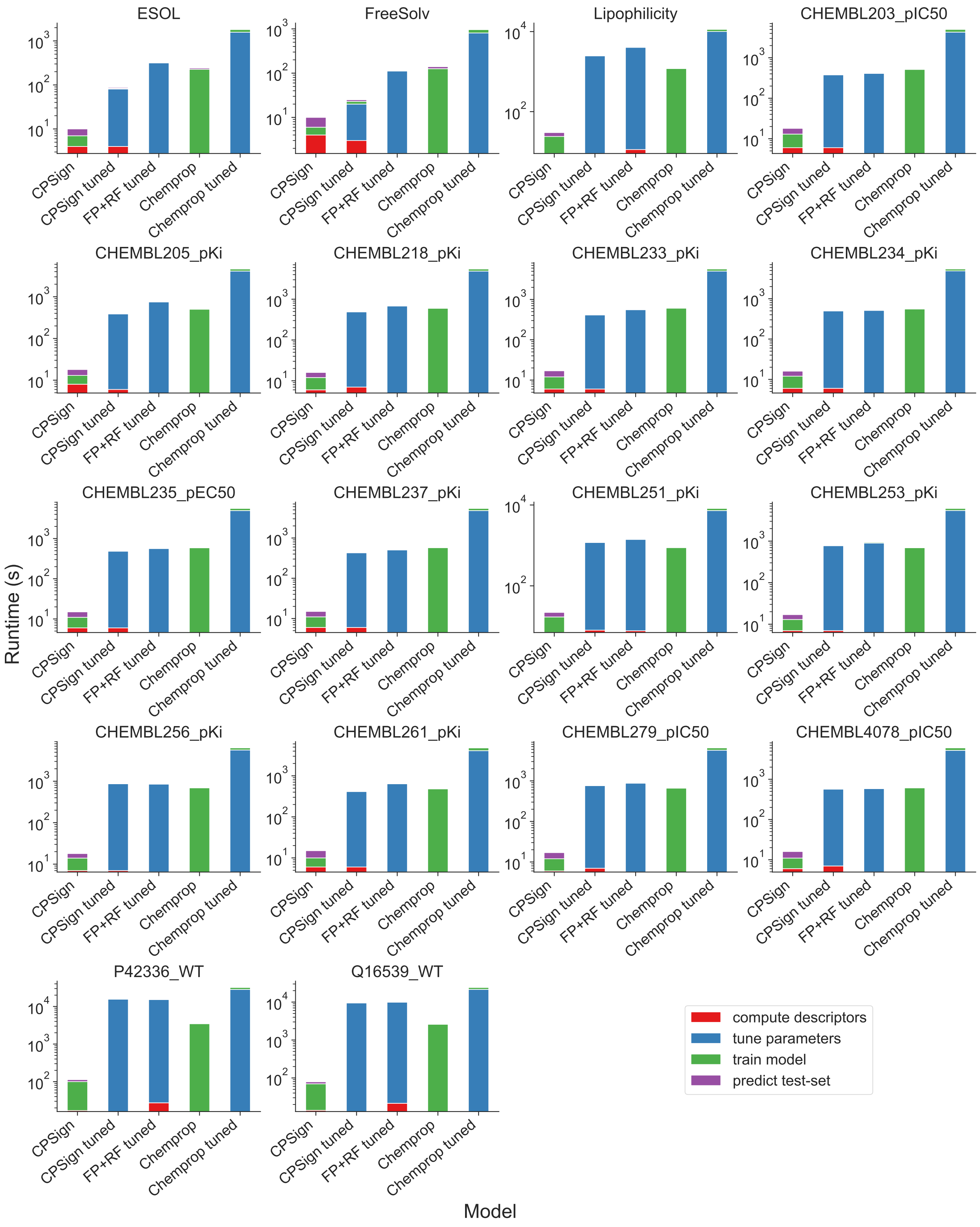

Figure 8: Runtime comparison for individual regression datasets, using a logarithmic y-axis. The relative runtimes are consistent across all runs, with only minor differences between the CPSign tuned, FP+RF tuned and Chemprop methods. Note that the two Chemprop methods do not contain a separate step for computing descriptors, which instead is included in the tuning and training steps.

Table 3: Grid of predicted atom contributions for ten recent cancer drugs, based on six of the regression datasets and using the CPSign method. Each image uses a blue-red coloring scheme, where blue indicate atoms part of features (substructures) contributing towards a lower predicted value, and vice versa for red. We can see that each model finds different substructures as the most important for their prediction (i.e., looking row-wise in the grid) and that the prediction intervals differ in their width between different drugs (i.e., looking column-wise). The prediction width is based on the estimated difficulty in predicting the drug based on the error model.

Lipophilicity	FreeSolv	ESOL
Larotrectinib<br>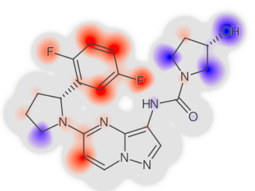<br>Prediction (*conf*=0.80):<br>(-0.911 ; 0.399)<br>Prediction width: 1.31	Larotrectinib<br>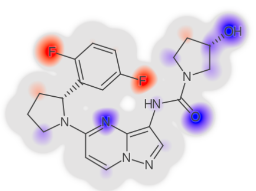<br>Prediction (*conf*=0.80):<br>(-2.71 ; -1.76)<br>Prediction width: 0.95	Larotrectinib<br>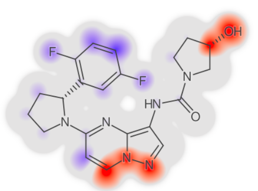<br>Prediction (*conf*=0.80):<br>(-0.139 ; 0.938)<br>Prediction width: 1.08
Entrectinib<br>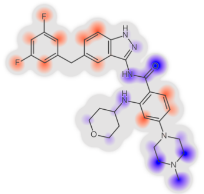<br>Prediction (*conf*=0.80):<br>(0.125 ; 1.47)<br>Prediction width: 1.35	Entrectinib<br>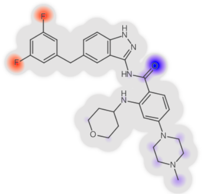<br>Prediction (*conf*=0.80):<br>(-2.22 ; -1.42)<br>Prediction width: 0.799	Entrectinib<br>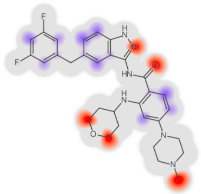<br>Prediction (*conf*=0.80):<br>(-1.56 ; -0.69)<br>Prediction width: 0.873
Ripretinib<br>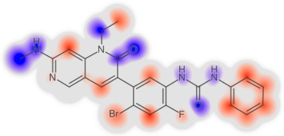<br>Prediction (*conf*=0.80):<br>(0.266 ; 1.36)<br>Prediction width: 1.09	Ripretinib<br>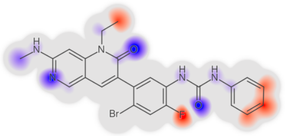<br>Prediction (*conf*=0.80):<br>(-2.05 ; -1.54)<br>Prediction width: 0.513	Ripretinib<br>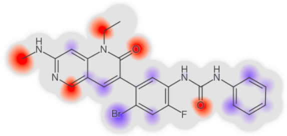<br>Prediction (*conf*=0.80):<br>(-1.37 ; -0.459)<br>Prediction width: 0.915
Tazemetostat<br>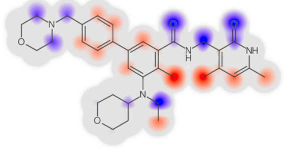<br>Prediction (*conf*=0.80):<br>(-0.0383 ; 1.26)<br>Prediction width: 1.3	Tazemetostat<br>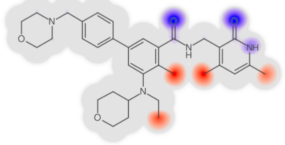<br>Prediction (*conf*=0.80):<br>(-2.63 ; -1.83)<br>Prediction width: 0.8	Tazemetostat<br>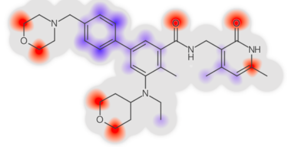<br>Prediction (*conf*=0.80):<br>(-1.48 ; -0.761)<br>Prediction width: 0.721
Tucatinib<br>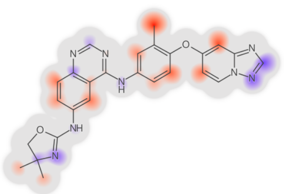<br>Prediction (*conf*=0.80):<br>(0.311 ; 1.64)<br>Prediction width: 1.33	Tucatinib<br>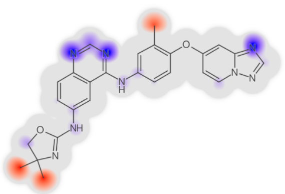<br>Prediction (*conf*=0.80):<br>(-1.85 ; -1.29)<br>Prediction width: 0.561	Tucatinib<br>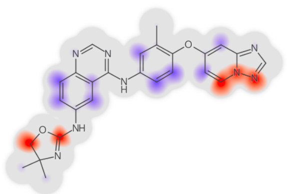<br>Prediction (*conf*=0.80):<br>(-1.14 ; 0.0225)<br>Prediction width: 1.16

Lipophilicity	FreeSolv	ESOL
Pexidartinib<br>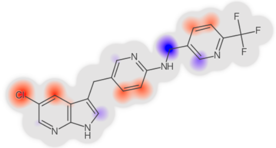<br>Prediction (*conf*=0.80):<br>(0.797 ; 1.88)<br>Prediction width: 1.08	Pexidartinib<br>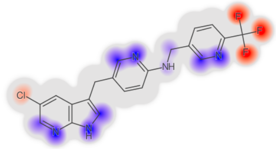<br>Prediction (*conf*=0.80):<br>(-1.18 ; -0.605)<br>Prediction width: 0.57	Pexidartinib<br>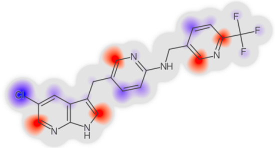<br>Prediction (*conf*=0.80):<br>(-0.758 ; -0.0513)<br>Prediction width: 0.707
Avapritinib<br>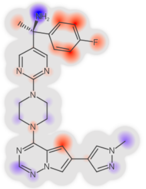<br>Prediction (*conf*=0.80):<br>(0.0372 ; 1.32)<br>Prediction width: 1.28	Avapritinib<br>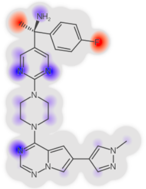<br>Prediction (*conf*=0.80):<br>(-2.07 ; -1.47)<br>Prediction width: 0.603	Avapritinib<br>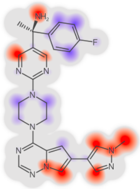<br>Prediction (*conf*=0.80):<br>(-0.61 ; 0.409)<br>Prediction width: 1.02
Capmatinib<br>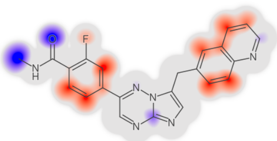<br>Prediction (*conf*=0.80):<br>(-0.301 ; 0.803)<br>Prediction width: 1.1	Capmatinib<br>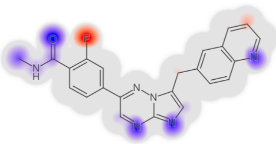<br>Prediction (*conf*=0.80):<br>(-2.54 ; -1.73)<br>Prediction width: 0.816	Capmatinib<br>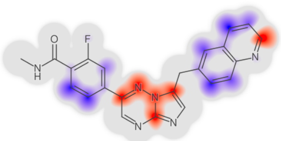<br>Prediction (*conf*=0.80):<br>(-0.83 ; 0.0402)<br>Prediction width: 0.87
Fedratinib<br>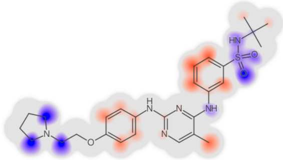<br>Prediction (*conf*=0.80):<br>(-0.721 ; 0.777)<br>Prediction width: 1.5	Fedratinib<br>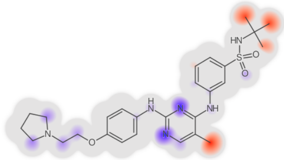<br>Prediction (*conf*=0.80):<br>(-2.04 ; -1.38)<br>Prediction width: 0.659	Fedratinib<br>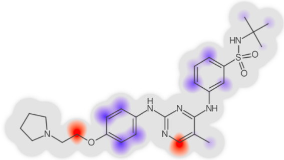<br>Prediction (*conf*=0.80):<br>(-1.04 ; -0.347)<br>Prediction width: 0.697
Methotrexate<br>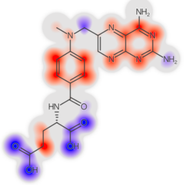<br>Prediction (*conf*=0.80):<br>(-3.83 ; -2.88)<br>Prediction width: 0.948	Methotrexate<br>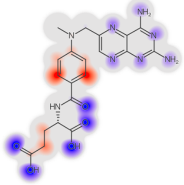<br>Prediction (*conf*=0.80):<br>(-4.76 ; -3.89)<br>Prediction width: 0.871	Methotrexate<br>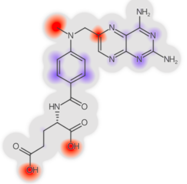<br>Prediction (*conf*=0.80):<br>(-0.21 ; 0.723)<br>Prediction width: 0.933

CHEMBL203_pIC50	CHEMBL235_pEC50	CHEMBL279_pIC50
Larotrectinib<br>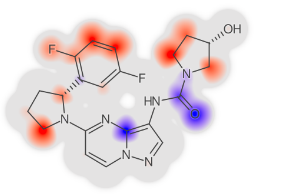<br>Prediction (*conf*=0.80):<br>(3.83 ; 6.31)<br>Prediction width: 2.48	Larotrectinib<br>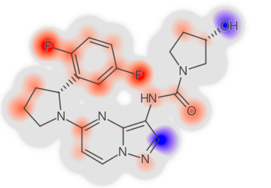<br>Prediction (*conf*=0.80):<br>(4.85 ; 5.44)<br>Prediction width: 0.591	Larotrectinib<br>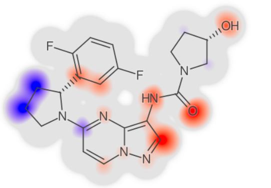<br>Prediction (*conf*=0.80):<br>(6.16 ; 8.53)<br>Prediction width: 2.36
Entrectinib<br>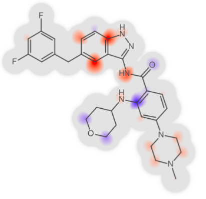<br>Prediction (*conf*=0.80):<br>(5.17 ; 6.72)<br>Prediction width: 1.55	Entrectinib<br>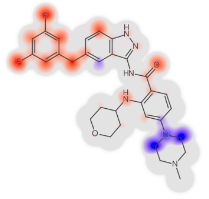<br>Prediction (*conf*=0.80):<br>(5.34 ; 6.44)<br>Prediction width: 1.1	Entrectinib<br>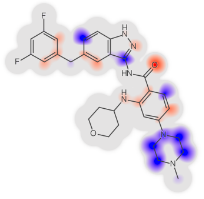<br>Prediction (*conf*=0.80):<br>(6.29 ; 7.28)<br>Prediction width: 0.988
Ripretinib<br>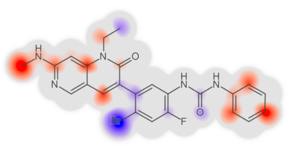<br>Prediction (*conf*=0.80):<br>(4.7 ; 6.55)<br>Prediction width: 1.85	Ripretinib<br>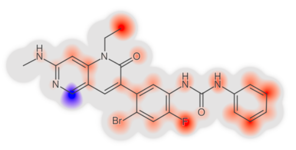<br>Prediction (*conf*=0.80):<br>(4.76 ; 6.34)<br>Prediction width: 1.58	Ripretinib<br>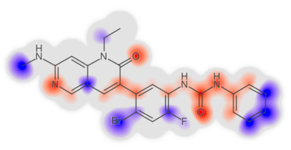<br>Prediction (*conf*=0.80):<br>(5.72 ; 8.35)<br>Prediction width: 2.64
Tazemetostat<br>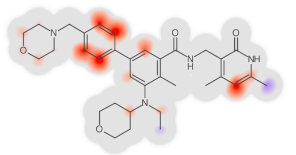<br>Prediction (*conf*=0.80):<br>(4.58 ; 6.77)<br>Prediction width: 2.19	Tazemetostat<br>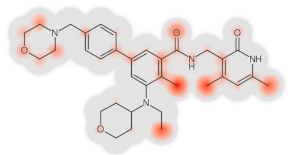<br>Prediction (*conf*=0.80):<br>(5.55 ; 7.31)<br>Prediction width: 1.76	Tazemetostat<br>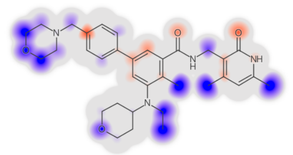<br>Prediction (*conf*=0.80):<br>(6.51 ; 8.04)<br>Prediction width: 1.53
Tucatinib<br>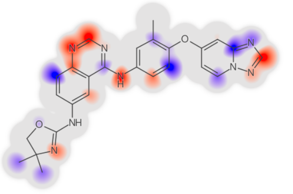<br>Prediction (*conf*=0.80):<br>(5.29 ; 7.54)<br>Prediction width: 2.25	Tucatinib<br>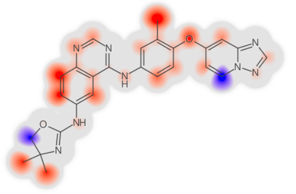<br>Prediction (*conf*=0.80):<br>(4.82 ; 6.51)<br>Prediction width: 1.69	Tucatinib<br>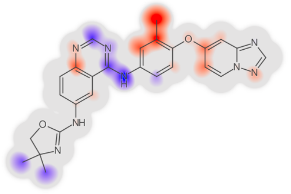<br>Prediction (*conf*=0.80):<br>(5.42 ; 7.33)<br>Prediction width: 1.91

CHEMBL203_pIC50	CHEMBL235_pEC50	CHEMBL279_pIC50
Pexidartinib<br>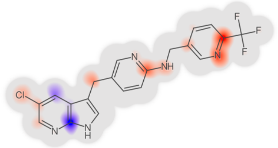<br>Prediction (*conf*=0.80):<br>(3.93 ; 5.93)<br>Prediction width: 2.01	Pexidartinib<br>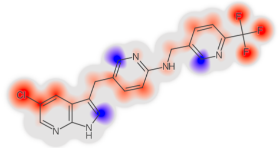<br>Prediction (*conf*=0.80):<br>(5.18 ; 6.94)<br>Prediction width: 1.76	Pexidartinib<br>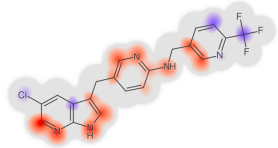<br>Prediction (*conf*=0.80):<br>(5.63 ; 7.19)<br>Prediction width: 1.56
Avapritinib<br>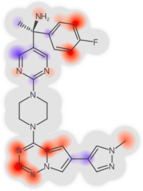<br>Prediction (*conf*=0.80):<br>(4.82 ; 6.89)<br>Prediction width: 2.07	Avapritinib<br>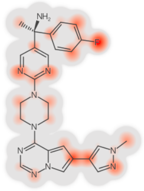<br>Prediction (*conf*=0.80):<br>(4.98 ; 5.68)<br>Prediction width: 0.694	Avapritinib<br>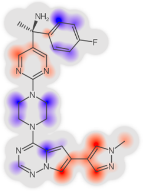<br>Prediction (*conf*=0.80):<br>(6.12 ; 7.36)<br>Prediction width: 1.25
Capmatinib<br>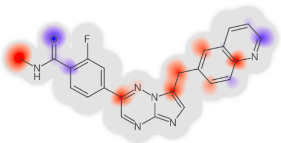<br>Prediction (*conf*=0.80):<br>(5.14 ; 7.06)<br>Prediction width: 1.92	Capmatinib<br>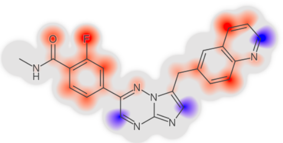<br>Prediction (*conf*=0.80):<br>(4.78 ; 6.38)<br>Prediction width: 1.6	Capmatinib<br>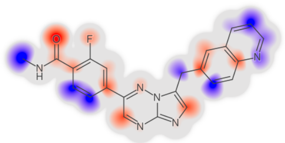<br>Prediction (*conf*=0.80):<br>(5.51 ; 7.7)<br>Prediction width: 2.19
Fedratinib<br>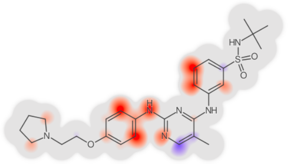<br>Prediction (*conf*=0.80):<br>(4.5 ; 6.24)<br>Prediction width: 1.74	Fedratinib<br>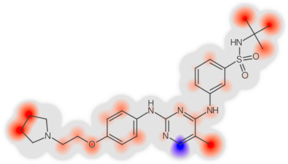<br>Prediction (*conf*=0.80):<br>(4.57 ; 6.28)<br>Prediction width: 1.71	Fedratinib<br>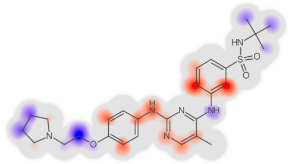<br>Prediction (*conf*=0.80):<br>(5.41 ; 7.39)<br>Prediction width: 1.98
Methotrexate<br>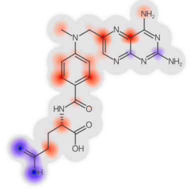<br>Prediction (*conf*=0.80):<br>(3.55 ; 5.1)<br>Prediction width: 1.55	Methotrexate<br>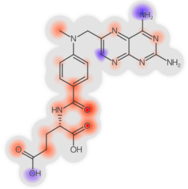<br>Prediction (*conf*=0.80):<br>(4.2 ; 5.82)<br>Prediction width: 1.61	Methotrexate<br>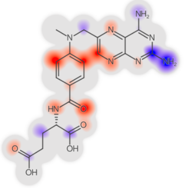<br>Prediction (*conf*=0.80):<br>(5.59 ; 7.54)<br>Prediction width: 1.95
